# Beneficial Effect of *Cissus quadrangularis* Linn. on Osteopenia Associated with Streptozotocin-Induced Type 1 Diabetes Mellitus in Male Wistar Rats

**DOI:** 10.1155/2014/483051

**Published:** 2014-04-07

**Authors:** Srinivasa Rao Sirasanagandla, Sreedhara Ranganath Pai Karkala, Bhagath Kumar Potu, Kumar M. R. Bhat

**Affiliations:** ^1^Department of Anatomy, Melaka Manipal Medical College, Manipal University, Madhav Nagar, Manipal, Karnataka 576104, India; ^2^Department of Pharmacology, Manipal College of Pharmaceutical Sciences, Manipal University, Manipal, Karnataka 576104, India; ^3^Department of Anatomy, College of Medicine and Medical Sciences, Arabian Gulf University, P.O. Box 26671, Bahrain; ^4^Department of Anatomy, Kasturba Medical College, Manipal University, Manipal, Karnataka 576104, India

## Abstract

Petroleum ether fraction of *Cissus quadrangularis* (*PECQ*) impact on the development of osteopenia in type 1 diabetic rat model has been evaluated. Diabetic rats were treated orally with two doses of * PECQ*. Another group of diabetic rats were treated with subcutaneous injection of synthetic human insulin. The cortical and trabecular bone thickness and bone strength were significantly decreased in diabetic rats. Treatment with two doses of * PECQ* significantly prevented these changes in diabetic rats. However, * PECQ* treatment (two doses) did not alter the glycemic levels in these diabetic rats. Increased levels of serum alkaline phosphatase (ALP), tartrate-resistant acid phosphatase (TRAP), and hydroxyproline were noted in diabetic rats when compared to normal control rats. The two doses of * PECQ* treatment further improved the serum ALP levels and significantly decreased the serum levels of TRAP and hydroxyproline. The effects of * PECQ *treatment on histological, biomechanical, and biochemical parameters are comparable to those of insulin. Since * PECQ* improves the bone health in hyperglycemic conditions by enhancing the cortical and trabecular bone growth and altering the circulating bone markers, it could be used as an effective therapy against diabetes-associated bone disorders.

## 1. Introduction

Diabetes mellitus (DM) is a combination of metabolic disorders characterized by impaired metabolism of carbohydrates, proteins, and fat resulting from insulin deficiency [1]. Skeletal disorders are common in diabetic patients, namely, reduced bone mineral content [2, 3], deranged calcium and phosphate levels, and altered bone metabolism [3–6]. Osteopenia [7], increased risk of fractures [8], and delayed fracture healing [9] are evident in these patients. Earlier, animal models have proved the association between osteopenic/osteoporotic changes and type 1 DM [10–15]. It has been demonstrated that the adverse effects of DM on bone tissue could be due to insulinopenia, bone microangiopathy, impaired regulation of mineral metabolism, and alterations in local factors that regulate bone remodeling [16, 17]. Dimensions of the femur such as weight, length, and diaphyseal width were found to be decreased in diabetic rats [18]. Furthermore, experimental studies have demonstrated that the mechanical strength of bones is reduced in diabetic rats [19–21]. Diabetes is also found to delay fracture healing and treatment with synthetic calcium phosphate or hydroxyapatite has been shown to have a positive effect on fracture healing [22–24]. It was also shown that the treatment with either insulin or 17-b estradiol (E2) can reverse the altered architecture of bones in diabetic rats and their effects were found to be similar [25]. Verhaeghe et al. have observed the positive effect of E2 against the metaphyseal trabecular bone damage in ovariectomized diabetic rats, when compared to nontreated control rats [26].

*Cissus quadrangularis *(*CQ*) is a climbing shrub, which belongs to the Vitaceae family. It is usually seen in hot climate in various states of India, Sri Lanka, Malaya, Java, and West Africa [27]. In Ayurveda, its usage in the treatment of bone fractures and swelling has been mentioned [28].* CQ* has been shown to have an ability to accelerate the healing of bone fracture [29]. Experimental animal models have proved the antiosteoporotic potential of ethanol, petroleum ether, and hexane fractions of the* CQ* [27, 30, 31]. A pharmacological study on* CQ* has shown the presence of phytoestrogen steroids [32]. Recently, the phytoestrogen-rich fraction separated from the* CQ* has been shown to have potent antiosteoporotic activity [33].* In vitro* study has shown that the petroleum ether fraction of* CQ* (*PECQ*) enhances the proliferation and differentiation of rat bone marrow mesenchymal stem cells [34]. Previously in our laboratory, the protective effect of* PECQ* on defective fetal skeletal ossification in maternal diabetes has been studied (ahead of print). Though the pharmacological effect of* CQ* on bone health has been studied extensively, no attempt has been made to study the efficacy of* CQ* on osteopenia associated with DM. In the presented study, we evaluated the effect of* CQ* against bone histology, biomechanical changes, and circulating bone markers in type 1 diabetic rats.

## 2. Material and Methods

### 2.1. Preparation of Plant Extract

The fresh stems of* CQ* were air-dried and grinded into powder. Using a soxhlet apparatus, 1.3 kg* CQ* powder was subjected to extraction with 95% ethyl alcohol. The ethanol extract obtained (125 g) was further suspended in water. Then, it was partitioned with petroleum ether (b.p. 60–90°C) solvent. The total yield (9.1% w/w) of* PECQ* was stored at 4°C until use. Every day fresh suspension of* PECQ *was prepared by using carboxymethyl cellulose (CMC).

### 2.2. Animals

In the present study, 3-month-old male Wistar rats (180–220 g weight) were used. After obtaining approval from the Institutional Animal Ethical Committee, rats were placed in the Central animal research facility of Manipal University according to guidelines of the Committee for the Purpose of Control and Supervision of Experiments on Animals (CPCSEA). Proper ventilation with temperature control was maintained on a 12 hr dark and 12 hr light schedule throughout the experiment. Rats were fed a standard balanced diet and water.

### 2.3. Induction of Diabetes and Treatments

After one week male Wistar rats were induced diabetes with streptozotocin (STZ) injection intraperitoneally (40 mg/kg body weight), which was dissolved in 0.1 M citrate buffer, pH 4.5. Control rats were injected 0.1 M citrate buffer. The blood glucose levels were analyzed seven days after injection using Glucometer (AccuChek Active). Animals demonstrating hyperglycemia (>250 mg/dL) were treated orally with* PECQ* at daily doses of 500 mg/kg and 750 mg/kg body weight. The dose was selected based on the previous study [30]. The diabetic rats in another group were injected subcutaneously twice daily with human synthetic insulin (INS) (Actrapid, Novo Nordisk India Pvt. Ltd., India), at a dose of 10 U/kg body weight. Treatment continued for 45 days.

### 2.4. Experimental Design

Experimental rats (*n* = 30) were allocated to 5 groups each containing 6 rats. Rats in normal control (NC) Group A received 0.5% CMC; Group B, diabetic control group (DC), received 0.5% CMC; Group C, the diabetic + CQ1 group (DC + CQ1), received 500 mg/kg body weight dose of* PECQ*; Group D, the diabetic + CQ2 group (DC + CQ2), received 750 mg/kg body weight dose of* PECQ*; and Group E, the diabetic + INS group (DC + INS), rats received INS. The blood glucose levels were analyzed at regular intervals of the experimental period. Following the completion of experiment, the animals were sacrificed under anesthesia by cervical dislocation. Before sacrificing the animals, blood was collected for estimation of serum ALP, TRAP, and hydroxyproline. Right femora were collected for histomorphometrical analysis of trabecular bone and cortical bone. Left femora were stored at −70°C for testing the biomechanical properties. Right tibia was collected for measuring the dry weight.

### 2.5. Histomorphometrical Analysis

Left femora were dissected and the soft tissue separated. Tissues were fixed in the PLP fixative for 24 hr at 4°C. Then, the femora were subjected to decalcification using EDTA-glycerine solution. After 20 days of complete decalcification, tissues were dehydrated and placed in paraffin wax. The longitudinal sections (5 *μ*m thickness) of the lower end of the femur were taken on rotary microtome and then processed for eosin and hematoxylin staining. The stained sections were used for analysis of the thickness of trabecular bone in the metaphyseal and epiphyseal regions, by using Olympus Cellsens Imaging Software (1.6 version, USA).

### 2.6. Measurement of Biomechanical Properties

The maximum flexor load was measured by a three-point bending test, using a Universal testing 3366 machine (Instron Corp, UK). Briefly, the left femora were brought to room temperature from −70°C and wiped with tissue paper. In the material testing machine, the bone was placed horizontally on two supports and load was applied in the middle of the shaft, at a speed of 5 mm/min until the bone was fractured.

### 2.7. Biochemical Analysis of Serum Bone Markers

ALP and TRAP levels were estimated by spectrophotometric method, using commercially available kits (Agappe diagnostics). Serum hydroxyproline levels were analyzed by Neuman and Logan method [35].

### 2.8. Dry Weight of the Tibia

Right tibia were collected and dissected free of soft tissue. Bone tissues were kept in a hot-air oven at 110°C for 48 hr and were weighed in digital balance as described previously [36].

### 2.9. Statistical Analysis

Results were expressed as the mean ± standard error of mean. Data was analyzed by using Graphpad Prism (version 5.1). One-way ANOVA followed by Bonferroni's multiple comparison test was used to evaluate differences between groups. Statistical significance was considered at *P* < 0.05.

## 3. Results

### 3.1. Effect of* PECQ* on Blood Glucose Levels

DC rats had hyperglycemia (>250 mg/dL) throughout the experiment. The two doses of* PECQ* treatment did not alter the blood glucose levels in diabetic rats when compared to diabetic nontreated rats (*P* > 0.05; Table 1). However, insulin treatment significantly decreased the blood glucose levels, when compared to DC group (*P* < 0.001; Table 1).

### 3.2. Effect of* PECQ* on Trabecular Bone in Epiphyseal Region

**Figure 1:**
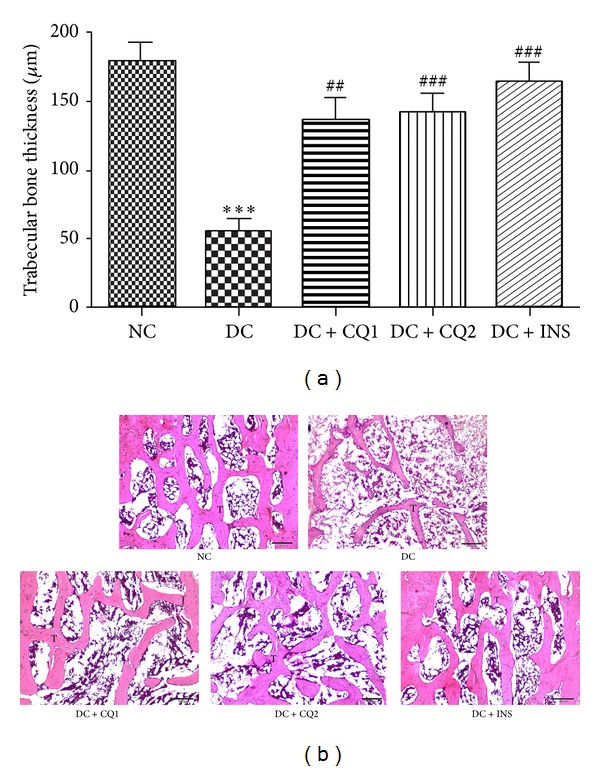
(a) Effect of* PECQ* on mean thickness of trabecular bone in the epiphyseal region. Significant decrease in the thickness of the trabecular bone was observed in the diabetic control (DC) rats when compared to normal control (NC) rats. However, diabetic rats treated with two different doses of* PECQ *(DC + CQ1 & DC + CQ2) or with insulin (DC + INS) showed a significant increase in bone thickness. ***P < 0.001 when compared to NC group; ^###^ *P* < 0.001, ^##^ *P* < 0.01 when compared to DC group. (b) Photomicrographs of trabecular bone in epiphyseal region. Thinner and reduced number of trabeculae can be seen in the diabetic control group (DC) when compared to normal control group (NC). Further treatment with two doses of* PECQ* (DC + CQ1, DC + CQ2) and insulin (DC + INS) improved the trabecular bone thickness. T: trabecular bone; H and E staining, scale bar: 200 *μ*m.

DC rats had thinner trabeculae in the epiphyseal region (*P* < 0.001; Figures 1(a) and 1(b)) when compared to NC rats suggesting that the hyperglycemia affects the normal bone architecture and leads to bone loss in the epiphyseal region. Treatment with* PECQ* significantly improved the trabecular bone thickness in the DC + CQ1 (*P* < 0.01; Figures 1(a) and 1(b)) and DC + CQ2 (*P* < 0.001; Figures 1(a) and 1(b)) groups when compared to DC rats. On the other hand, metabolic control with INS significantly prevented the bone loss in diabetic rats (*P* < 0.001; Figures 1(a) and 1(b)) when compared to DC rats.

### 3.3. Effect of* PECQ* on Trabecular Bone in Metaphyseal Region

**Figure 2:**
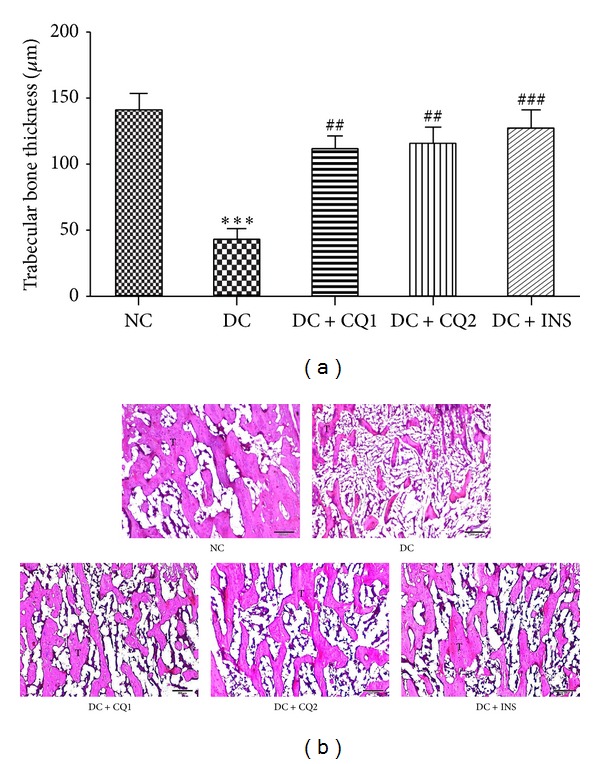
(a) Effect of* PECQ *on mean thickness of trabecular bone in the metaphyseal region. Thickness of the trabecular bone significantly decreased in diabetic control (DC) rats when compared to normal control (NC) rats. However, treatment with two different doses of* PECQ* (DC + CQ1 and DC + CQ2) or with insulin (DC + INS) showed a significant increase in the bone thickness, when compared to diabetic nontreated rats. ***P < 0.001 when compared to NC group; ^###^ *P* < 0.001, ^##^ *P* < 0.01 when compared to DC group. (b) Photomicrographs of the trabecular bone in metaphyseal region. Thinner, disrupted, and reduced number of trabeculae were observed in the diabetic control group (DC) when compared to the normal control group (NC). Further treatment with two doses of* PECQ* (DC + CQ1, DC + CQ2) and insulin (DC + INS) improved the trabecular bone growth. T: trabecular bone; H and E staining, scale bar: 200 *μ*m.

Thinner trabeculae were observed in the DC rats (*P* < 0.001; Figures 2(a) and 2(b)) when compared to NC rats, suggesting that hyperglycemia also affects the bone growth in the metaphyseal region. Treatment with two doses of* PECQ *significantly improved the trabecular bone thickness in DC + CQ1 and DC + CQ2 groups (*P* < 0.01; Figures 2(a) and 2(b)) when compared to diabetic nontreated rats. INS treatment also significantly improved the bone thickness in DC + INS rats (*P* < 0.001; Figures 2(a) and 2(b)) when compared to diabetic nontreated rats.

### 3.4. Effect of* PECQ *on Cortical Bone

**Figure 3:**
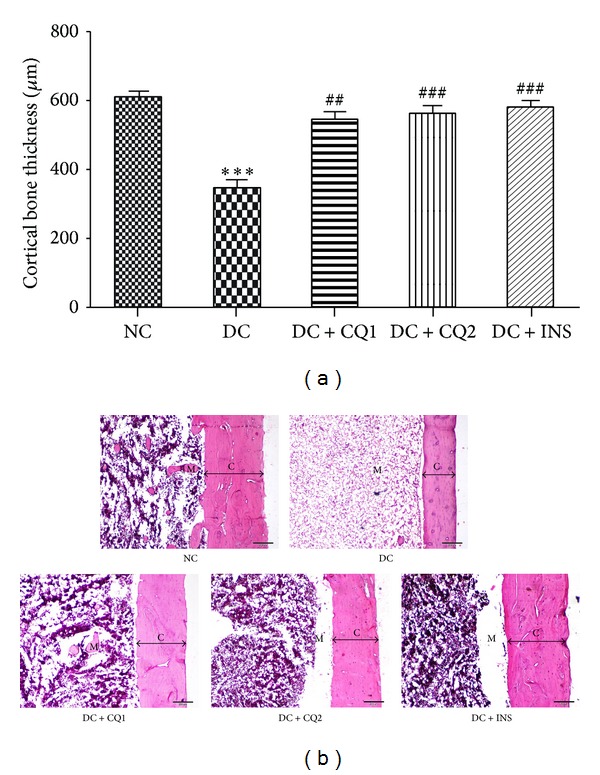
(a) Effect of* PECQ* on mean thickness of cortical bone. Thickness of the cortical bone significantly decreased in diabetic control (DC) rats when compared to normal control (NC) rats. Treatment with two different doses of* PECQ* (DC + CQ1 and DC + CQ2) or with insulin (DC + INS) significantly increased the bone thickness in diabetic rats when compared to nontreated diabetic rats. ***P < 0.001 when compared to NC group; ^###^ *P* < 0.001, ^##^ *P* < 0.01 when compared to DC group. (b) Photomicrographs of cortical bone. Thickness of the cortical bone was significantly less in the diabetic control group (DC) when compared to normal control group (NC). Further treatment with two doses of the* PECQ* (DC + CQ1, DC + CQ2) and insulin (DC + INS) improved the cortical bone growth. C: cortical bone; M: medullary cavity; H and E staining, scale bar: 200 *μ*m.

The thickness of cortical bone significantly decreased in the DC group (*P* < 0.001; Figures 3(a) and 3(b)) when compared to NC rats, indicating the effect of hyperglycemia on cortical bone loss.* PECQ* treatment significantly improved the cortical bone thickness in the DC + CQ1 (*P* < 0.01; Figures 3(a) and 3(b)) and DC + CQ2 (*P* < 0.001; Figures 3(a) and 3(b)) groups when compared to DC rats. INS treatment also significantly improved the cortical bone thickness in diabetic rats (*P* < 0.001; Figures 3(a) and 3(b)) when compared to diabetic nontreated rats.

### 3.5. Effect of* PECQ *on Biomechanical Strength

**Figure 4:**
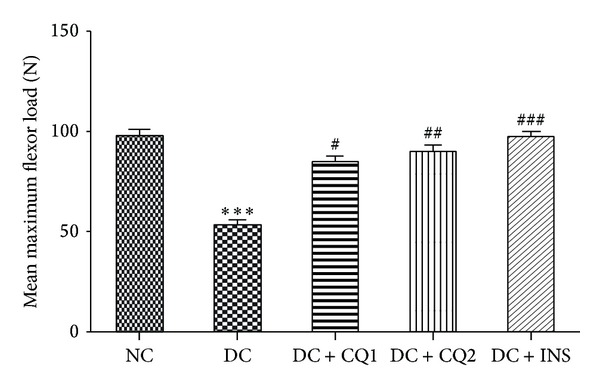
Effect of* PECQ* on mean maximum flexor load of femur. Mean maximum flexor load significantly decreased in diabetic control (DC) rats when compared to normal control (NC) rats. Treatment with two different doses of* PECQ *(DC + CQ1 and DC + CQ2) or with insulin (DC + INS) significantly increased the mean maximum flexor load in diabetic rats when compared to DC rats. ***P < 0.001 when compared to NC group; ^###^ *P* < 0.001, ^##^ *P* < 0.01, and ^#^ *P* < 0.05 when compared to DC group.

Mean maximum flexor load (N) required to produce break in the femur of NC, DC, DC + CQ1, DC + CQ2, and DC + INS groups was 96.53 ± 5.37, 53.2 ± 5.03, 76.47 ± 4.4, 81.42 ± 6.24, and 91.53 ± 4.79 newtons, respectively. Mean maximum flexor load was significantly less in the diabetic nontreated rats (*P* < 0.001; Figure 4), when compared to nondiabetic control rats. Further, mean maximum flexor load was significantly more in DC + CQ1 (*P* < 0.05), DC + CQ2 (*P* < 0.01), and DC + INS (*P* < 0.001) groups when compared to DC rats (Figure 4).

### 3.6. Effect of* PECQ* on Dry Weight of Tibia

**Figure 5:**
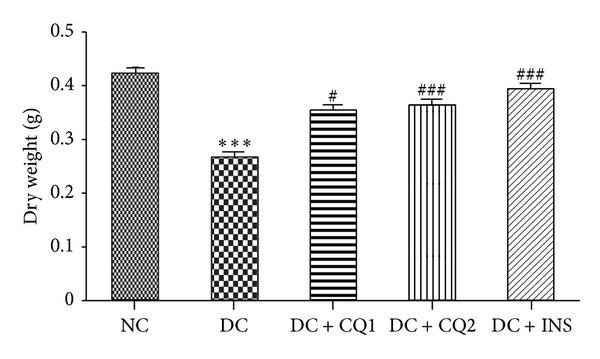
Effect of* PECQ* on dry weight of the tibia. Dry weight of the tibia significantly decreased in diabetic control (DC) rats when compared to normal control (NC) rats. Treatment with two different doses of* PECQ* (DC + CQ1 and DC + CQ2) or with insulin (DC + INS) significantly increased the dry weight in diabetic rats when compared to DC rats. ***P < 0.001 when compared to NC group; ^###^ *P* < 0.001, ^#^ *P* < 0.05 when compared to DC group.

Dry weight of the tibia measured in NC, DC, DC + CQ1, DC + CQ2, and DC + INS groups was 0.42 ± 0.019, 0.26 ± 0.017, 0.35 ± 0.013, 0.36 ± 0.021, and 0.39 ± 0.022 grams, respectively. Bone weight was significantly decreased in the DC rats (*P* < 0.001; Figure 5), when compared to NC rats. Dry weight was significantly increased in all the treated groups, DC + CQ1 (*P* < 0.05), DC + CQ2 (*P* < 0.05), and DC + INS (*P* < 0.001), when compared to DC rats (Figure 5).

### 3.7. Effect of* PECQ* on ALP, TRAP, and Hydroxyproline

Serum ALP levels were significantly increased in diabetic nontreated animals (*P* < 0.001; Table 2) when compared to NC group. Increased ALP levels confirm that diabetes induces bone damage. Serum ALP levels further increased in DC + CQ1 (*P* < 0.001), DC + CQ2 (*P* < 0.001), and DC + INS (*P* < 0.001) groups when compared to NC group (Table 2). This result shows that both* PECQ *and metabolic control with INS enhance the bone formation and mineralization process in hyperglycemic conditions. When compared to NC group, serum levels of TRAP (*P* < 0.001; Table 2) and hydroxyproline (*P* < 0.01; Table 2) were significantly increased in diabetic rats. Serum TRAP is a biomarker of osteoclast activity and hydroxyproline is considered as an end product of collagen degradation. The increased levels of these two proteins indicate the excessive bone resorption in the diabetic rats. Further, all the treatments significantly decreased the serum TRAP activity in the DC + CQ1 (*P* < 0.05), DC + CQ2 (*P* < 0.01), and DC + INS (*P* < 0.01) groups in comparison to diabetic nontreated rats (Table 2). Similarly, the hydroxyproline levels were also significantly decreased in the DC + CQ1 (*P* < 0.05), DC + CQ2 (*P* < 0.05), and DC + INS (*P* < 0.01) groups in comparison to DC group (Table 2).

## 4. Discussion 

Results of the present study showed that* PECQ* treatment is effective against type 1 DM- induced histological, biomechanical, and biochemical changes in the bone. Further, these results are comparable to the effects of insulin treatment. Unlike insulin,* PECQ* did not reduce the blood glucose levels in the diabetic rats indicating that* PECQ* has shown its effect through mechanisms other than the glycemic control.

The association between type 1 DM and osteoporosis has been accepted widely both experimentally [37, 38] and clinically [16, 39]. Based on the existing data, it is uncertain that reduced bone mass in diabetic rats is either due to defective bone formation or due to reduced bone growth [40]. Previous studies have reported the histological changes in both cortical bone [13, 41, 42] and trabecular bone [43, 44] in diabetic animals. Our results are consistent with those of earlier studies wherein the diabetic rats showed a marked reduction in the thickness of both cortical and trabecular bones. The diabetic rats also had decreased dry weight of the bone compared to healthy animals. Bone strength depends on the integrity of the two components of bone: cortical and trabecular bone. Previous studies on the effect of diabetes on bone strength have reported conflicting data. In few reports bone strength is increased [20, 21]; meanwhile it is reduced in others [13, 42]. Our results indicate that diabetic rats seem to have lower bone strength.

It has been hypothesized that inflammation plays a role in the pathology of diabetes- induced bone complications [45]. Cytokines such as IL-6, IL-1*β*, and TNF-*α* are known for their involvement in the process of bone loss in diabetes [46]. LT-*β*, IL-6, IFN-*γ*, and TNF-*α* were found to increase in the diabetic bone [45]. Anti-inflammatory activity of* CQ *has been shown by previous studies [47, 48]. Ethanol fraction of* CQ* has been shown to decrease the serum levels of the proinflammatory cytokines TNF-*α*, IL-1*β*, and IL-6 in ovariectomized mice [48]. The positive effect of* PECQ* on bone loss in the diabetic state could be due to its anti-inflammatory property. However, experimental evidence is required to confirm effect of* PECQ* on both bone and serum cytokines levels in diabetic state.

Hyperglycemia is known to alter the antioxidant defense by increasing the polyol pathway flux, rate of formation of the ROS, and glucose-derived advanced glycosylation end products [49]. Previous studies have confirmed the association between oxidative stress and the development of osteopenia in DM [50, 51]. ROS is known to stimulate bone resorption by altering the function of osteoclasts [52]. Bai et al., have observed that oxidative stress can inhibit the differentiation of osteoblast cells [53]. Previous studies have demonstrated antioxidant and free radical scavenging potential of* CQ* both* in vitro* and* in vivo* [54, 55]. Hence, beneficial effects of* PECQ* against bone damage in diabetic rats can be correlated to its antioxidant properties.

Endocrine factor such as insulin-like growth factor-1 (IGF-1) signaling is found to be downregulated in both humans and animal models with type I DM [56, 57]. Decreased bone mineral density and altered osteoblast differentiation are seen with low serum levels of IGF-1 [58, 59]. Further, it has been demonstrated that improving the serum IGF-1 levels can prevent bone loss in diabetic rats [60]. Muthusami et al. studied the effect of ethanol extract* CQ *on IGF system components and found that* CQ* can enhance the mRNA expression of IGF-IR, IGF-I, and IGF-II [61]. Based on the above facts, we hypothesize that* PECQ* could have prevented the bone loss in diabetic rats by increasing the expression of IGF system components. However, further studies are required to confirm the effect of the* PECQ* on IGF system components in hyperglycemic conditions.

Earlier studies have demonstrated the beneficial effect of synthetic estrogen E2 against the bone loss in hyperglycemic state [25, 26]. The positive effect of* PECQ* on bone changes in diabetic rats observed in the present study could be due to the phytoestrogen steroids present in it [32, 33], which may increase the bone formation and/or accelerate bone growth. Alterations in the mineral metabolism and bone remodeling factors are claimed to be the possible mechanism of diabetes-induced osteoporosis [17]. It has been observed that altered bone turnover in diabetes is usually associated with the changes in serum ALP, TRAP, and hydroxyproline activities [25]. In the present study, ALP levels were significantly increased in diabetic rats. Serum ALP is considered as a biomarker of osteoblast activity. The increased ALP levels in the diabetic rats indicate the compensatory mechanism of the body against diabetes-induced bone damage. Administration of two doses of* PECQ* and INS showed further increase in the ALP levels compared to diabetic group. This result indicates that* PECQ* enhances osteoblast proliferation and thus facilitates the bone formation. An* in vitro* study has demonstrated the stimulatory effect of E2 on differentiation of bone marrow mesenchymal stem cells, in hyperglycemic conditions [62]. The beneficial effect of* PECQ* on ALP activity could be due to estrogen-mimicking action of phytoestrogen steroids present in it [32, 33].

Compared to normal control group, serum TRAP levels are significantly increased in diabetic animals. Serum TRAP is a biomarker of osteoclast activity. The increased levels of this protein indicate that the reduced bone mass in the diabetic rats is also due to excessive bone resorption. With respect to serum TRAP activity, our results are consistent with previous findings [25]. However, Waud et al. have observed normal TRAP activity in the experimental diabetic animals [37]. In another study on patients with type I diabetes, TRAP activity was found to be low [63]. Administration of* PECQ* and INS significantly decreased the TRAP levels compared to diabetic animals. The observed effect of the* PECQ* on the TRAP activity could be due to the direct action of the phytoestrogen steroids present in it [32, 33]. This can be explained based on the fact that estrogen can accelerate the apoptosis of matured osteoclast cells [64].

Serum hydroxyproline is a breakdown product of collagen. In the present study, the hydroxyproline levels were significantly increased in the diabetic rats indicating the inhibitory effect of diabetes on bone collagen. This result is consistent with that of the previous study [25]. Treatment with* PECQ* and INS significantly decreased the serum hydroxyproline levels. Gopalakrishnan et al. in their* in vitro* study demonstrated the positive effect of E2 on mineralization and histochemical staining for collagen in the bone marrow stromal cells [62]. The positive effect of the* PECQ* could be due to the estrogen-like action of phytoestrogen steroids [32, 33] on collagen formation.

## 5. Conclusion

Preliminary results of the present study indicate that* PECQ* is effective in improving histological, biomechanical, and biochemical changes of bone in diabetic rats. Though exact mechanism of action of* PECQ *has not been ascertained, the observed effect of* PECQ* could be due to its osteogenic, antioxidant, and anti-inflammatory properties. However, in this context, extensive studies are required to confer the exact mechanism of* PECQ* on bone cells in hyperglycemic conditions.

## Figures and Tables

**Table 1:** Effect of *PECQ* on blood glucose levels (mg/dL) in diabetic rats.

Groups	Day 0	Day 5	Day 15	Day 25	Day 35	Day 45
NC	89 ± 5.71	97.16 ± 5.02	92.5 ± 3.51	95.5 ± 4.58	99.83 ± 4.86	105.33 ± 5.14
DC	294.5 ± 9.66***	351.16 ± 23.5***	390 ± 18.44***	394 ± 19.52***	398 ± 26.51***	381 ± 21.59***
DC + CQ1	305 ± 13.8	342.66 ± 21.12	374.5 ± 14.49	379.5 ± 19.97	372.83 ± 23.65	375 ± 25.91
DC + CQ2	308.66 ± 13.48	346.33 ± 14.25	364.66 ± 22.08	365 ± 19.81	379 ± 26.61	378.5 ± 17.49
DC + INS	292.66 ± 9.05	102.83 ± 6.35^$$$^	109.5 ± 6.14^$$$^	99.16 ± 4.63^$$$^	117.16 ± 5.31^$$$^	103.83 ± 6.1^$$$^

***P < 0.001 when compared to NC group. ^$$$^ *P* < 0.001 when compared to DC group.

**Table 2:** Effects of *PECQ* on serum bone markers in diabetic rats.

Groups	ALP (U/L)	TRP (U/L)	Hydroxyproline (*μ*g/mL)
NC	96.45 ± 5.93	6.40 ± 0.29	0.233 ± 0.008
DC	188.7 ± 7.02***	8.52 ± 0.19***	0.285 ± 0.007**
DC + CQ1	217.3 ± 9.4***	6.90 ± 0.24^$^	0.250 ± 0.007^$^
DC + CQ2	223.3 ± 13.21***	6.74 ± 0.40^$$^	0.249 ± 0.009^$^
DC + INS	229.1 ± 9.75***	6.57 ± 0.37^$$^	0.239 ± 0.009^$$^

***P < 0.001, **P < 0.01 when compared to NC group. ^$$^ *P* < 0.01, ^$^ *P* < 0.05 when compared to DC group.
